# The Prognostic Value of the C-reactive Protein/Prognostic Nutritional Index Ratio in Stage III and IV Laryngeal Cancer Patients Treated with Radiotherapy

**DOI:** 10.7759/cureus.4648

**Published:** 2019-05-12

**Authors:** Jie Fu, Xiaojing Yang

**Affiliations:** 1 Radiation Oncology, Shanghai Jiao Tong University Affliated Sixth People’s Hospital, Shanghai, CHN

**Keywords:** laryngeal cancer, crp/pni, prognosis

## Abstract

Objective

Patients with advanced laryngeal cancer have a poor prognosis. The purpose of this work was to analyze the connection between clinical factors and survival and to try to identify survival prognostic factors.

Methods

Sixty-one laryngeal cancer patients received radiotherapy. All of their clinicopathologic material was gathered from a retrospective review of the medical records and subjected to further analysis. We examined the prognostic significance of the C-reactive protein (CRP)/prognostic nutritional index (PNI) ratio.

Results

We assessed the CRP and PNI levels before radiotherapy. An analysis of overall survival (OS) of patients with high CRP/PNI was markedly shorter than for those patients with a low CRP/PNI (P=0.010). Multivariable analysis showed that a high CRP/PNI ratio was a disadvantageous independent prognostic marker.

Conclusions

The data show that CRP/PNI may be used as a prognostic indicator for laryngeal cancer patients treated with radiotherapy.

## Introduction

Laryngeal cancer is a common malignancy of the head and neck. The incidence of laryngeal cancer is notably higher in men than in women [1]. The most common pathological type of larynx cancer, making up the majority of cases, is squamous cell cancer [2]. The treatment of laryngeal cancer usually depends on surgical treatment and radiotherapy. Radiotherapy plays an important role in patients at advanced clinical stages [3]. Although treatment methods continue to improve, the therapeutic effect is still not satisfactory. The establishment of a simple and convenient marker system to help determine the prognosis of patients with laryngeal cancer would be of great significance.

There are reports showing that C-reactive protein (CRP) levels are associated with inflammatory responses. High expression of CRP indicates a poor outcome in patients with malignant tumors, including laryngeal cancer [4]. In addition, it has also been proposed that the inflammatory response is pathogenic to cancer-related malnutrition [5-6]. Malnutrition, in turn, is associated with poor overall state, shortened survival, and increased mortality in cancer patients [7]. The prognostic nutrition index (PNI) is used to evaluate the outcomes of patients with malignant tumors, such as esophageal cancer [8], lung cancer [9], and gastric cancer [10]. In recent work, CRP/PNI showed a prognostic ability in esophageal cancer patients [8] and fracture surgery patients [11]. Raised CRP/PNI predicted poor prognosis. However, there has been no research that has shown the relationship between the prognostic value of CRP/PNI and laryngeal cancer.

The object of this research was to evaluate the significance of CRP/PNI and survival in laryngeal cancer patients. We looked into the impact of CRP/PNI on overall survival (OS) in laryngeal cancer and contrasted the predictive value of platelet count, CRP, PNI, leukocytosis, lymphocyte counts, anemia, and CRP/PNI.

## Materials and methods

Patients and data collection

We retrospectively analyzed 61 patients with laryngeal cancer who were treated in our department from January 2009 to June 2014. The main standards for inclusion are as follows: (i) all of the patients had a pathological diagnosis of laryngeal cancer; and (ii) none of the patients had undergone chemotherapy or radiotherapy before. Patients were excluded for the subsequent criteria: (i) acute infection occurring within two weeks; (ii) other blood system diseases; or (iii) incomplete data. The following clinical parameters were recorded: gender, age at diagnosis, differentiation, tumor stage, tumor, nodes, and metastases (TNM) stage, lymph node metastasis (LNM), and smoking history. The laboratory characteristics of blood reports, including platelet count, CRP, PNI, leukocytosis, lymphocyte counts, and anemia, were performed before radiotherapy. The following formula was calculated for PNI: 0.05 total lymphocyte count (/mm3) + 10 × serum albumin (g/dL) [12]. Authors have access to information that could identify individual participants during or after data collection. Ethical approval for the use of patient tissues was provided by the Ethics Committee of Shanghai Sixth People’s Hospital (Shanghai, China). Informed consent was obtained from all patients.

Statistical analysis

The data analysis was performed using the SPSS statistical software package (Version 20.0; IBM Corporation, Armonk, NY, USA). The Spearman rank correlation survival analysis was used to study the correlation between CRP and PNI. The survival analysis was tested for significance using Kaplan-Meier curves and log-rank tests. Univariable Cox regression analyses were performed using death as the outcome. The Cox proportional hazards model was used for multivariate analysis, to evaluate the relationship between CRP/PNI and other prognostic factors and OS. We calculated the hazard ratio and 95% confidence interval. P<0.05 was considered statistically significant.

## Results

Patient characteristics

Sixty-one patients undergoing radiotherapy were involved in this study. Each patient’s features are shown in Table 1. The median age of the patients was 57.2 years (±7.12) and 59 patients (96.7%) were male. The patients’ tumor stage distributed from T1 to T4, and there were 27 (44.3%) patients with negative N-stage. Among the 61 patients, 46 (75.4%) had a history of smoking. The cell types in our data showed 59 patients (96.7%) with squamous cell carcinoma and the others were adenocarcinoma.

**Table 1:** Clinicopathological characteristics of laryngeal cancer patients

Characteristics	No. of patients (%)
Gender	
Male	59 (96.7)
Female	2 (3.3)
Age, years	
Mean	57.2
SD	7.12
Differentiation	
Well/moderate	32 (52.5)
Poor/undifferentiation	29 (47.5)
Tumor stage	
T1	5 (8.2)
T2	9 (14.8)
T3	26 (42.6)
T4	21 (34.4)
Tumor, node, metastases (TNM) stage	
III	35 (57.4)
IV	26 (42.6)
Lymph node metastasis (LNM)	
Yes	27 (44.3)
No	34 (55.7)
Smoking history	
Yes	46 (75.4)
No	15 (24.6)
Pack-years	
≤30	26 (42.6)
>30	35 (57.4)
Survival status	
Dead	36 (59.0)
Alive	25 (41.0)

CRP/PNI ratio as a prognostic marker of survival

**Figure 1:**
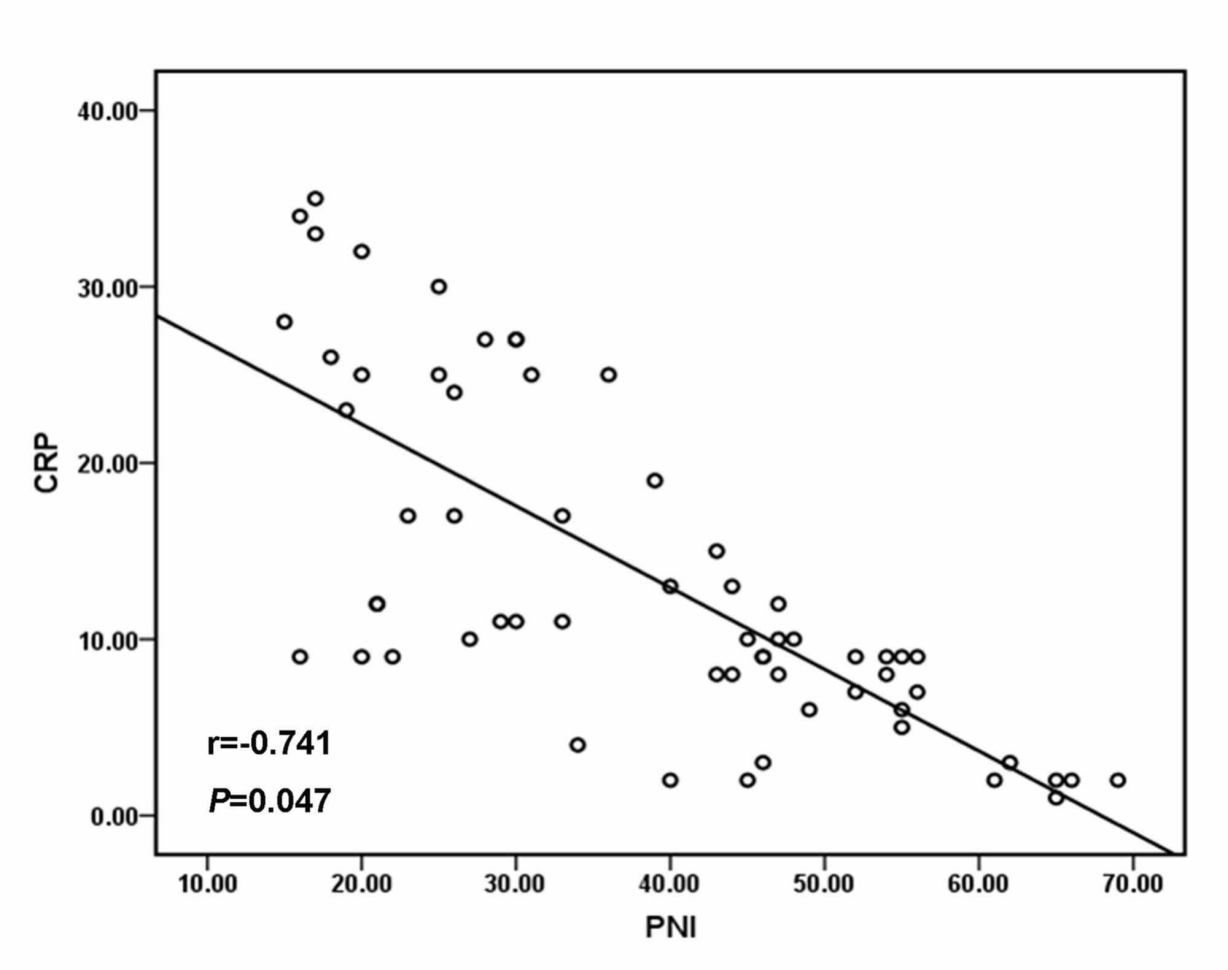
Pearson correlation A significant negative correlation between CRP and PNI (r=-0.741, p=0.047) CRP: C-reactive protein, PNI: prognostic nutritional index

**Figure 2:**
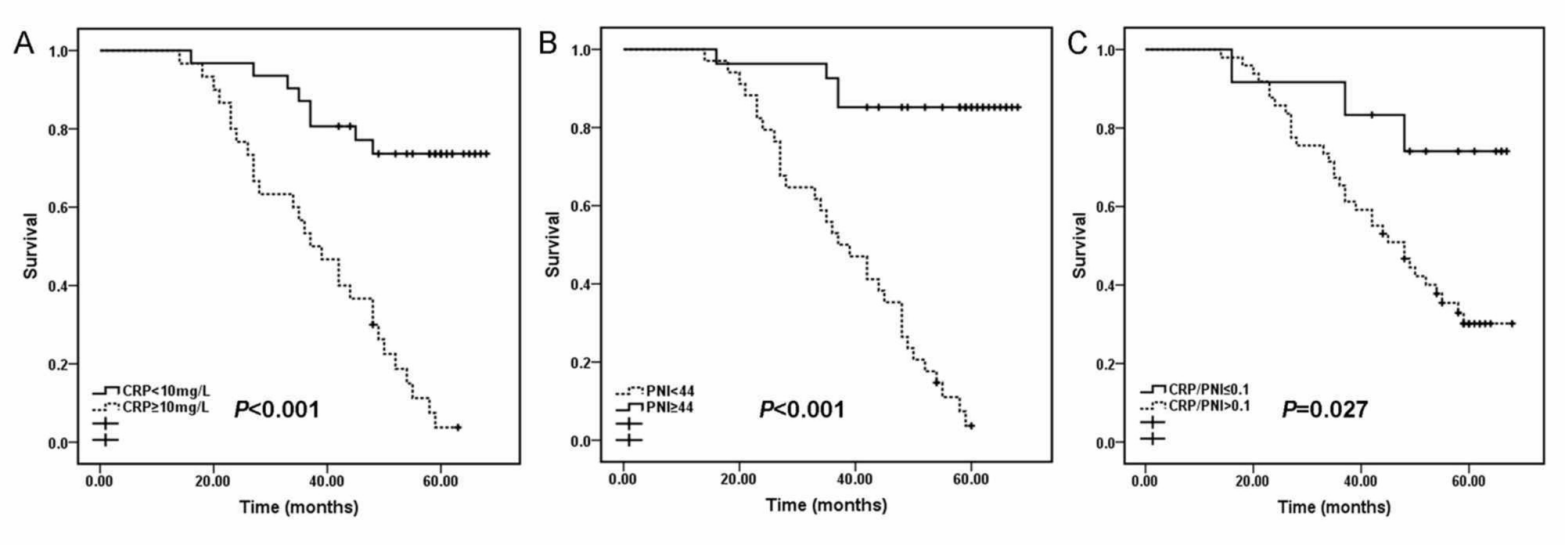
Kaplan-Meier survival curves stratified by CPR, PNI, and CRP/PNI ratio A and B: patients with elevated CRP (p<0.001) or decreased PNI (p<0.001) were associated with decreased five-year survival, respectively. C: Patients with a CRP/PNI ratio of ≤0.10 had better survival than patients with a CRP/PNI ratio of >0.10 (p<0.001). CRP: C-reactive protein, PNI: prognostic nutritional index

The mean values of CRP and PNI were 13.65 ± 7.34 mg/L and 38.42 ± 8.26, respectively. There was a negative correlation between PNI and CRP with a coefficient of -0.741 (Figure 1; p=0.047). We show the relationship between CRP/PNI ratios and clinical properties in Table 2. The cut-off value of the CRP/PNI ratio was 0.10 according to the receiver operating characteristic (ROC) analysis. According to this value, patients were separated into the CRP/PNI ratio ≤0.10 group and the CRP/PNI ratio >0.10 group. Twelve patients (19.7%) had a CRP/PNI ratio of ≤0.10 and 49 patients (80.3%) had a CRP/PNI ratio of >0.10. The CRP/PNI ratio was associated with CRP (p<0.001), PNI (p<0.001), platelet count (p=0.049), hypoalbuminemia (p=0.032), lymphocyte count (p=0.046), and survival (p=0.010). However, no relationship was observed between the CRP/PNI ratio and other factors, such as comorbidities, leukocytosis, and anemia. Kaplan-Meier survival curves with high CRP (p<0.001, Figure 2A) or low PNI (p<0.001, Figure 2B) were associated with poor survival. In addition, patients with a low proportion of CRP/PNI and a high CRP/PNI ratio displayed obvious separation (p=0.027, Figure 2C).

**Table 2:** CRP/PNI ratio and clinicopathological parameters CRP = C-reactive protein, PNI = prognostic nutritional index, * p<0.05 is considered significant

	Total cases (n)	CRP/PNI ratio		p-value
		≤ 0.10	> 0.10	
Age (years)	57±7	56±3	58±6	0.317
Men	59	11	48	0.752
Female	2	1	1	
CRP, mg/L				
<10	31	12	19	﹤0.001*
≥10	30	0	30	
Platelet count, /mm^3^				
<214	33	3	30	0.049*
≥214	28	9	19	
Leukocytosis				
Yes	40	10	30	0.190
No	21	2	19	
Anemia				
Yes	31	6	25	0.949
No	30	6	24	
Lymphocyte counts, /mm^3^				
<1673	33	4	29	0.046*
≥1673	28	8	20	
Hypoalbuminemia				
Yes	32	4	28	0.032*
No	29	8	21	
PNI				
<44	34	1	33	﹤0.001*
≥44	27	11	16	
Survival status				
Dead	36	3	33	0.010*
Alive	25	9	16	

Survival and prognostic factor analysis

The five-year OS value was 41.0%. The univariate and multivariate analyses of OS in all the patients are shown in Table 3 and Table 4, respectively. In the univariate analysis, PNI (p<0.001), CRP (p=0.04), lymphocyte counts (p<0.001), leukocytosis (p=0.015), hypoalbuminemia (p=0.037), and CRP/PNI (p=0.010) were found to have a significant impact on OS. In the multivariate analysis, we found PNI (p=0.009) and CRP/PNI (p=0.018) to be predictive factors for survival. Collectively, these data show that a high CRP/PNI ratio predicts poor prognosis for laryngeal cancer.

**Table 3:** Survival status and clinicopathological parameters in 61 specimens CRP = C-reactive protein, PNI = prognostic nutritional index, * p<0.05 is considered significant

	Total cases (n)	Survival status		p-value
		Dead=36	Alive=25	
Age (years)	57±7	58±3	56±2	0.065
Men	59	36	23	0.071
Female	2	0	2	
CRP, mg/L				
<10	31	8	23	﹤0.001*
≥10	30	28	2	
Platelet count, /mm^3^				
<214	33	33	10	0.075
≥214	28	13	15	
Leukocytosis				
Yes	40	19	21	0.015*
No	21	17	4	
Anemia				
Yes	31	14	17	0.037
No	30	22	8	
Lymphocyte counts, /mm^3^				
<1673	33	12	21	﹤0.001*
≥1673	28	24	4	
Hypoalbuminemia				
Yes	32	25	7	0.037*
No	29	11	18	
PNI				
<44	34	32	2	﹤0.001*
≥44	27	4	23	
CRP/PNI				
≤ 0.10	12	3	9	0.010*
> 0.10	49	33	16

**Table 4:** Contribution of various potential prognostic factors to survival by Cox regression analysis in 61 specimens CRP = C-reactive protein, PNI = prognostic nutritional index, CI = confidence interval, Statistical analyses were performed by the log-rank test, * p<0.05 is considered significant

	Hazard ratio	95 % CI	P
Age (years)	1.578	0.870~2.518	0.570
Sex	1.503	0.358~3.611	0.145
CRP, mg/L	2.577	0.729~7.961	0.052
PNI	0.143	0.076~8.047	0.009*
Platelet count, /mm^3^	1.937	0.722~5.741	0.198
Leukocytosis	1.332	0.544~4.902	0.731
Anemia	1. 476	0.786~4.516	0.415
Lymphocyte counts, /mm^3^	2.726	1.9013~7.457	0.062
Hypoalbuminemia	3.114	1. 731~5.920	0.326
CRP/PNI	2.375	2.104~11.101	0.018*

## Discussion

Therapeutic effects on patients with advanced laryngeal cancer have not significantly improved in the past two decades [13]. Our study aimed to discover a novel, comprehensive, and economical index for prognostic prediction. In this research, we showed that the CRP/PNI ratio was a predictor of advanced laryngeal cancer patients.

The occurrence and development of cancer are closely related to inflammation [14]. CRP is an important inflammatory marker whose levels rise in response to infection or trauma [15]. It has been reported that high levels of CRP can promote tumorigenesis and lead to poor prognosis in ovarian cancer [16-17], colon cancer [18], glioblastoma [19], and other conditions. CRP can increase vascular growth factors levels and interleukins in the peripheral blood, thereby promoting the formation of tumor blood vessels [20]. In the present study, patients with lower CRP level (≤10.0 mg/L) had better survival rates than patients with CRP >10.0 mg/L (74.2% vs. 6.7%, p<0.001). Unfortunately, in multivariate analysis, there was no evidence that CRP is an independent prognostic factor (p= 0.052).

More than 20% of cancer patients are reported to die of malnutrition rather than the tumor itself [21]. Laryngeal cancer patients are more prone to malnutrition due to the tumor mass and dysphagia [22]. PNI is calculated from serum albumin and lymphocyte counts. Albumin is closely related to inflammatory responses in cancer patients [23]. Lymphocytes regulate the immunologic damage caused by tumor cells and take a significant part in the immune response [24]. PNI is related to the prognosis of cancer patients with esophageal carcinoma [25], non-small cell lung cancer [26], ovarian cancer [27], and hepatocellular carcinoma [7]. PNI is considered an independent marker of poor prognosis in cancer patients. Consistent with previous results, PNI was found to be an independent prognostic factor in our results (p=0.009).

A single indicator may be affected by many factors; the ratio of CRP and PNI may reduce this effect. The ratio of CRP/PNI is more predictive than CRP or PNI by themselves. Thus, in the present research, we first studied the importance of CRP/PNI in evaluating the prognosis of laryngeal cancer patients. Compared with a CRP/PNI ratio of >0.10, patients with a CRP/PNI ratio of ≤0.10 had a better survival prognosis (p=0.001). In a multivariate analysis, the CRP/PNI ratio is an important non-independent prognostic factor (p=0.018). Consistent with previous results in esophageal cancer, our data show that the CRP/PNI ratio could be a predictor of laryngeal cancer.

This work has some specific deficiencies. First, our results are consistent with the predecessor’s outcomes. We found the CRP/PNI ratio to be a prognostic indicator. However, inﬂammatory marker leukocytosis is also related to survival in our results. The CRP/PNI ratio should be assessed together with leukocytosis in laryngeal cancer patients in further studies. What is more important is that this is a retrospective analysis of only 61 advanced laryngeal cancer cases. The sample size of the study is not sufficient. In future studies, a larger cohort of patients is required. If conditions permit, we hope to conduct a prospective study to enable a better evaluation of prognostic factors for laryngeal cancer patients.

In summary, our study identified a CRP/PNI ratio of >0.10 to be a signature indicator for the outcomes of advanced laryngeal cancer. The potential role of the CRP/PNI ratio and other indicators in laryngeal cancer remains to be investigated at a broader and deeper level.

## Conclusions

The purpose of our work is to analyze the connection between clinical factors and survival and to try to identify survival prognostic factors. We examined the prognostic significance of the C-reactive protein (CRP)/prognostic nutritional index (PNI) ratio. CRP/PNI may be used as a prognostic indicator for laryngeal cancer patients treated with radiotherapy.
